# Ubiquitin Specific Protease 29 Functions as an Oncogene Promoting Tumorigenesis in Colorectal Carcinoma

**DOI:** 10.3390/cancers13112706

**Published:** 2021-05-31

**Authors:** Arun Pandian Chandrasekaran, Bharathi Suresh, Neha Sarodaya, Na-Re Ko, Seung-Jun Oh, Kye-Seong Kim, Suresh Ramakrishna

**Affiliations:** 1 Department of Biomedical Science, Graduate School of Biomedical Science and Engineering, Hanyang University, 222 Wangsimni-ro, Seongdong, Seoul 04763, Korea; arunbio@hanyang.ac.kr (A.P.C.); bharathi@hanyang.ac.kr (B.S.); nsarodaya21@hanyang.ac.kr (N.S.); 2 Biomedical Research Center, Asan Institute for Life Sciences, Seoul 05505, Korea; nare.ko@amc.seoul.kr; 3 Asan Medical Center, Department of Nuclear Medicine, University of Ulsan College of Medicine, 88 Olympic-ro 43-gil, Songpa, Seoul 05505, Korea; 4 College of Medicine, Hanyang University, Seoul 04763, Korea

**Keywords:** colorectal carcinoma, deubiquitinating enzymes, ubiquitin specific protease, CRISPR-Cas9, oncogenesis, DNA damage, mouse models

## Abstract

**Simple Summary:**

Among other cancers, colorectal carcinoma (CRC) is one of the foremost causes of death worldwide. The mortality rate of those having CRC has increased dramatically in the past few years. Identification of novel regulatory molecules contributing to the progression of CRC remains a focus of significant interest. The oncogenic role of USP29 has recently been explored in a few cancer types. However, evidence concerning the expression of USP29 in other cancers is currently lacking. We identified that USP29 is highly expressed in CRC and may contribute to the progression of CRC. Depletion of USP29 in HCT116 by CRISPR-Cas9 system reduced the growth of cancer cells. Furthermore, our data suggests that USP29 knockdown reduced the tumor volume of mouse xenograft models. Future investigations are required to validate the outcome of USP29-targted therapy in patients having CRC.

**Abstract:**

Colorectal carcinoma is the third foremost cause of cancer-related deaths and accounts for 5.8% of all deaths globally. The molecular mechanisms of colon cancer progression and metastasis control are not well studied. Ubiquitin-specific protease 29 (USP29), a deubiquitinating enzyme, is involved in the occurrence and development of wide variety of cancers. However, its clinical significance and biological roles in colorectal carcinoma (CRC) remain unexplored. In this research, we observed that the rate of USP29 overexpression was higher in colon cancer patient tissues relative to its corresponding normal tissues. CRISPR-Cas9-mediated depletion of USP29 triggered DNA double strand breaks and delayed cell-cycle progression in HCT116 cells. We also demonstrated that USP29 depletion hampers the colony formation and increases apoptosis of HCT116 cells. USP29 knockdown significantly decreased CRC cell proliferation in vitro. Depletion of USP29 in HCT116 cells substantially reduced the tumor volume of mouse xenografts. In conclusion, our study shows that elevated expression of USP29 promotes malignancy in CRC, suggesting that USP29 could be a promising target for colon cancer therapy.

## 1. Introduction

Colorectal carcinoma (CRC) is the third most common form of cancer among males and the second-most commonly diagnosed cancer in females. Despite the development of systematic and comprehensive therapies, the mortality rate of CRC is still about 35% [1], primarily as a result of recurrences and metastases [2]. The molecular mechanisms underlying CRC initiation and progression urgently need to be understood to enable early diagnosis and treatment of CRC. Therefore, the discovery of new molecules that can serve as biomarkers for CRC is of great importance.

Deubiquitinating enzymes (DUBs) mediate the removal of ubiquitin from targeted ubiquitylated substrates to regulate their activities. DUBs control the expression, localization, and activity of numerous proteins and participate in various cellular processes, including the growth, cell cycle, signal transduction, and apoptosis [3,4]. Moreover, growing body of evidence suggests that the dysfunction of the DUB system can degrade tumor suppressors and elevate oncoprotein levels [5,6]. DUBs also play a crucial role in cancer initiation and progression [7]. Accumulating evidence suggests that DUBs play a prominent role in tumorigenesis at multiple levels [8]. Approximately 100 DUBs that regulate different functions have been reported in humans, and they are expressed in various tissues and organs. The ubiquitin-specific proteases (USPs) is known to be the largest subfamily [9]. USPs contain different functional domains, such as zinc finger and ubiquitin-binding motifs, indicating that each USP can cooperate with different proteins and be involved in unique cellular processes [10]. It has been reported that the altered expression and mutation of USPs are closely linked with various human cancers [7].

USPs are cysteine proteases that are responsive to various electrophiles and have been targeted as therapies for various cancers [11]. Therefore, it is highly desirable to characterize which USPs are critical to each cancer type to enable precision cancer treatment. For the current study, we analysed the prognostic value of USP29 in CRC using a publicly available database. We then demonstrated that USP29 is an oncogene associated with high-grade CRC tumors.

## 2. Results

### 2.1. USP29 Is Highly Upregulated in Clinical Human Colon Cancer Tissues and Promotes Cell Proliferation

**Figure 1:**
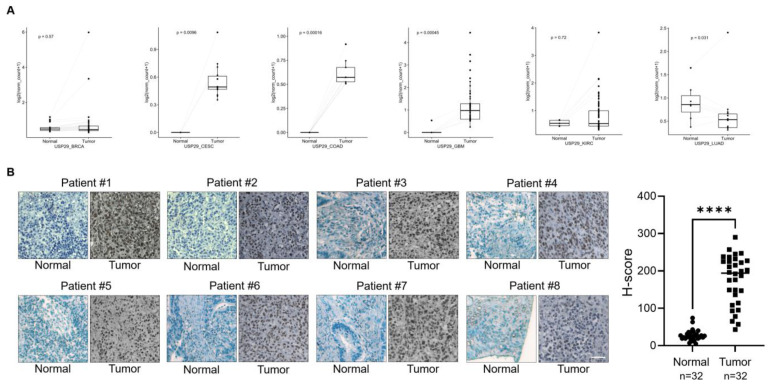
USP29 is upregulated in human colon cancer. (**A**) Box plot showing the expression patterns of USP29 in normal vcompared to tumor tissues of human colon cancer patients using TCGA RNAseq data. *p* < 0.05 is considered statistically significant. (**B**) Representative immunohistochemical staining of endogenous USP29 in human colon cancer and corresponding normal tissues. All IHC tissues were quantified by an *H* score. **** *p* < 0.0001 and Scale bar = 30 µm.

Previous researchers reported the functions of USP29 and its ability to regulate cancer-related proteins [12,13,14]. We sought for the clinical implications of USP29 expression in different cancer tissues. Using a publicly available database, we first investigated the expression level of USP29 in several cancer types. We observed that multiple cancer tissues had higher USP29 expression than the corresponding normal tissues (Figure 1A). However, significantly higher expression of USP29 (*p* = 0.00016) was observed in COAD patients than in other cancer cohorts (Figure 1A), strongly suggesting that USP29 might be associated with the progression of CRC. To investigate the expression of USP29 in human colon cancer tissues, we performed an immunostaining analysis in a tissue microarray (TMA) containing colon cancer tissues (*n* = 32) and corresponding normal tissues (*n* = 32) obtained from the ISU Abxis TMA. We found that USP29 was highly upregulated in colon cancer tissue when compared to its corresponding normal tissue (Figure 1B). The patient details for all 32 tumor samples were listed in Table S1.

**Figure 2:**
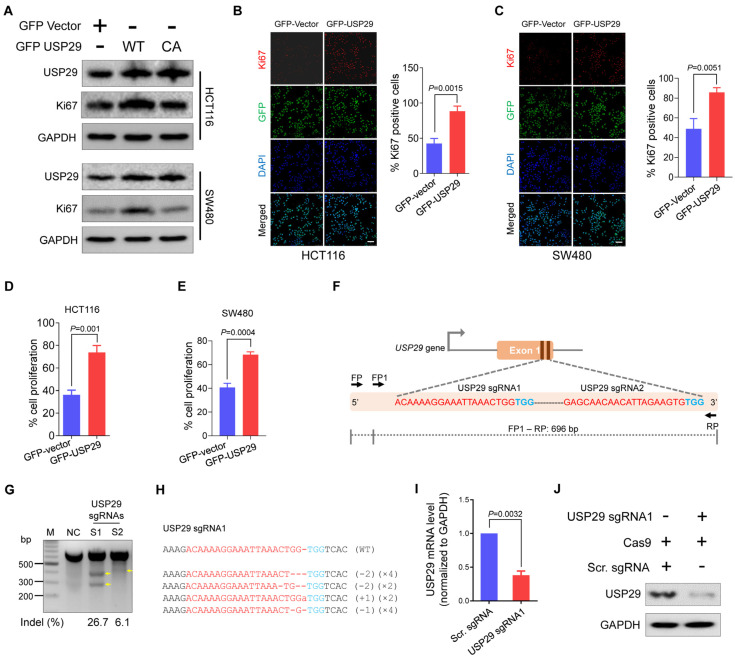
Overexpression of USP29 promotes cell proliferation, and USP29-depleted HCT116 cells were generated using CRISPR-Cas9. (**A**) HCT116 and SW480 cell lines were transfected with either GFP-vector, GFP USP29, or GFP-USP29 C294A (CA) and examined by western blot analysis using the indicated antibodies and GAPDH was used as a loading control. (**B**,**C**) Immunofluorescence analysis of Ki67 expression (red fluorescence), GFP vector or GFP-USP29 (green fluorescence), and nuclei (blue fluorescence) in the presence and absence of USP29 in HCT116 (**B**) and SW480 (**C**) cells. Scale bar = 50 µm. (**D**,**E**) CCK-8 cell proliferation assay showing the effect of USP29 in the HCT116 and SW480 human colon cancer cell lines. (**F**) Schematic representation of the sgRNA design strategy at exon 1 of the *USP29* gene. sgRNA sequences are in red and PAM sequences are denoted in blue. The PCR amplicon is indicated by the dotted line, along with the expected size (696 bp). (**G**) T7E1 assay to validate the knockout efficiencies of the designed sgRNAs in HCT116 cells (**H**) USP29 depletion as confirmed by Sanger sequencing. The sgRNA recognition site and PAM sequences are represented in red and blue, respectively. (**I**,**J**) The efficiency of USP29 depletion by sgRNA1 was validated by qPCR (**I**) and Western blot analysis (**J**). The number of deleted bases and the occurrence of sequences (e.g., ×1, ×2 or ×3 indicate the number of each sequence) are given in the parentheses on the right. Data in (**B**–**E**,**I**) are expressed as the mean ± standard deviation of three independent trials. Statistical analysis was evaluated by Student’s *t*-test, and *p*-values are as indicated.

To strengthen our observations, we analyzed the Ki67 expression, a marker for cell proliferation [15] using western blot in different colon cancer cells (HCT116 and SW480). Upon overexpression of USP29, the Ki67 expression level increased significantly compared with the vector control whereas catalytic inactive mutant of USP29 (USP29CA) did not alter the Ki67 expression (Figure 2A). The above results indicated that the DUB activity of USP29 is required for the proliferation of colon cancer cells. We next checked the interactions between endogenous USP29 and Ki67 in HCT116 cells. Our results showed that USP29 and Ki67 are not binding each other (Figure S1), suggesting that the expression of USP29 increases Ki67 expression in colon cancer cells without interacting with Ki67. We then immunostained HCT116 and SW480 in the presence of USP29 and analyzed Ki67 expression. Compared with the vector control, USP29 overexpression induces more proliferation in both HCT116 and SW480 cells (*p* = 0.0204 and *p* = 0.0141, respectively) (Figure 2B,C). We further used a CCK-8 assay kit to perform a cell proliferation assay in HCT116 and SW480 cells. USP29 overexpression significantly increased cell proliferation, compared with the vector controls, in HCT116 and SW480 cells (*p* = 0.001 and *p* = 0.0004, respectively) (Figure 2D,E). These findings suggest that USP29 could be an oncogene and promotes the proliferation of colon cancer.

### 2.2. Generation of USP29-Depleted Cells Using CRISPR-Cas9 System

Given that USP29 promotes the proliferative behavior of colon cancer cells, we examined its role in the canonical DNA damage and cell-cycle progression of HCT116 cells by generating USP29 knockout using the CRISPR-Cas9 system. Two sgRNAs specific to exon 1 of the *USP29* gene were designed (Figure 2F). These sgRNAs were previously designed and tested for their cutting efficiency in HEK293 cells [9]. The T7E1 assay was used to validate the cleavage efficiency of the sgRNAs targeting of the *USP29* gene in HCT116 cells. As shown in Figure 2G, sgRNA1 targeting USP29 showed cleavage efficiency at a significantly higher indel percentage than sgRNA2. To further confirm the *USP29* gene disruption, transfected cell pools of sgRNA1 targeting the *USP29* gene were subjected to Sanger sequencing.

The sequencing results demonstrated that sgRNA1 targeting USP29 displayed out-of-frame mutations (Figure 2H). The USP29-depleted HCT116 cells were subjected to qPCR and Western blot analysis to validate the knockdown effect of USP29. As shown in Figure 2I,J, USP29 was significantly downregulated compared to scrambled sgRNA control at mRNA and protein levels.

### 2.3. USP29 Depletion Induces DNA Damage and Delays Cell-Cycle Progression

**Figure 3:**
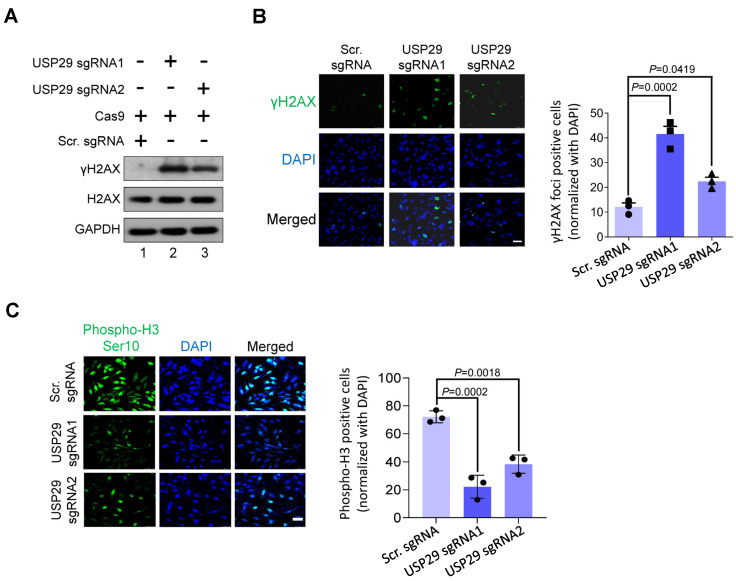
USP29 depletion triggers DNA damage and delays cell-cycle progression**.** (**A**) HCT116 cells were transfected with the indicated plasmids and analyzed by western blotting using indicated antibodies. GAPDH was used as a loading control. (**B**) γH2AX foci formation assay was performed to measure the DNA double strand breaks in HCT116 cells. Green, γH2AX; blue, nucleus stained with DAPI. Scale bar = 50 µm. The right panel shows the γH2AX-positive cells normalized with DAPI. (**C**) HCT116 cells were transfected with the indicated plasmids and immunostained with phospho-H3 (Ser10) antibody. The cells expressing phospho-H3 (green fluorescence) were counted and normalized with DAPI (blue fluorescence). Scale bar = 50 μm. The right panel shows the calculated data, presented as the phospho-H3-positive cells. Data in (**B**,**C**) are presented as the mean ± standard deviation of three independent trials. *p*-values were determined by one-way ANOVA followed by Tukey’s post hoc test.

To investigate whether the loss of USP29 function in HCT116 causes DNA damage, we checked the expression of a canonical DNA double strand break marker, γH2AX. As shown in Figure 3A, the loss of USP29 in HCT116 cells significantly elevated the expression of the γH2AX protein compared with the scrambled sgRNA control. It is noteworthy that sgRNA1 targeting USP29 induced more γH2AX protein expression than either sgRNA2 or the scrambled sgRNA control (Figure 3A, lane 2). Consistent with these results, USP29 knockouts displayed more robust γH2AX foci formation than the control HCT116 cells (Figure 3B). We next examined the role of USP29 in regulating the cell-cycle progression of HCT116 cells. To that end, we performed phospho-histone H3 (Ser10) immunostaining to analyze phosphorylated histone H3 in the mitotic chromosomes [16]. Consistent with the previous results, a reduction in the expression of phospho-histone H3 was observed in the absence of USP29 compared with the scrambled sgRNA control (Figure 3C). Therefore, our findings point to USP29 as being critical for cell-cycle progression in the HCT116 cell line.

### 2.4. USP29-Depleted Cells Undergo Apoptosis

**Figure 4:**
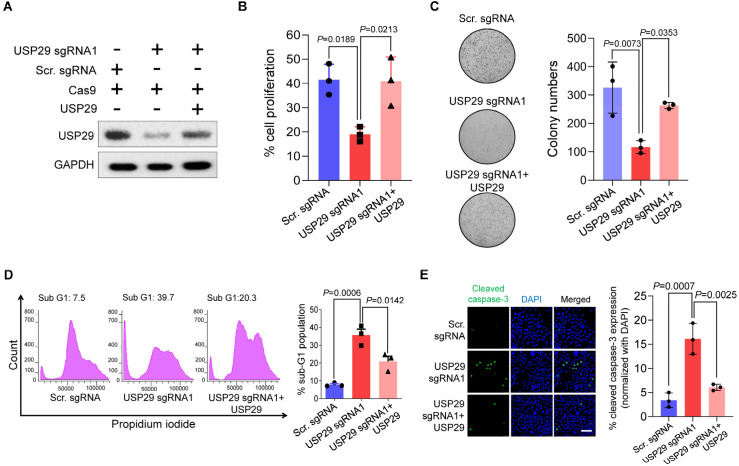
USP29-depleted HCT116 cells undergo apoptosis. (**A**) HCT116 cells were transfected with the indicated plasmids and Western blot analysis was performed with USP29-specific antibody. GAPDH was used as a loading control. (**B**) HCT116 cells were transfected with the indicated plasmids and subjected to a cell proliferation assay using a CCK-8 kit. (**C**) HCT116 cells were transfected with the indicated plasmids and subjected to a colony formation assay. The colonies were maintained for 14 days and stained with crystal violet to measure colony formation. The colony number quantified from the soft agar assay are presented in the right panel. (**D**) HCT116 cells were transfected with the indicated plasmids and stained with PI. Flow cytometry was performed to assess the apoptotic population. (**E**) HCT116 cells were transfected with the indicated plasmids and subjected to an immunofluorescence assay to analyze the expression of cleaved caspase-3. Scale bar = 50 µm. Quantitative result for cleaved caspase-3-positive cells are represented in the right panel. Data in (**B**–**E**) are presented as the mean ± standard deviation of three independent trials. *p*-values were calculated using one-way ANOVA followed by Tukey’s post hoc test.

To examine the oncogenic function of USP29 in colon cancer, USP29-depleted HCT116 cells and USP29-depleted cells reconstituted with USP29 were used in the following experiments and the transfected samples were confirmed by Western blot analysis (Figure 4A). USP29 depletion produced a pronounced decrease in cell proliferation compared with the scrambled sgRNA control (*p* = 0.0189) (Figure 4B). Reconstitution of USP29 in the USP29-depleted cells restored the proliferative behavior of the HCT116 cells (*p* = 0.0213) (Figure 4B). To corroborate those results, we subjected the USP29-depleted HCT116 cells to assays examining apoptosis and carcinogenesis-related activity. First, we performed an anchorage-independent colony formation assay in the presence and absence of USP29. USP29 depletion produced a significant reduction in colony numbers compared with the scrambled sgRNA control (*p* = 0.0073), and reconstitution of USP29 increased the colony numbers compared with the USP29-depleted group (*p* = 0.0353) (Figure 4C). Next, we used PI staining to analyze the apoptotic population by directly measuring DNA content in the sub-G1 phase. The results show a profound increase in the sub-G1 population when USP29 is depleted (*p* = 0.0006) (Figure 4D). Reconstituting USP29 in the USP29-depleted cells impeded apoptosis in HCT116 cells (*p* = 0.0142) (Figure 4D).

It has been reported that caspase-3 is a cysteine-aspartic acid protease that is cleaved and initiates apoptosis in cancer cells [17]. Therefore, we evaluated the levels of cleaved caspase-3 expression in HCT116 cells in the presence and absence of USP29. We found that USP29 depletion increased the expression of cleaved caspase-3 compared with the scrambled sgRNA control (*p* = 0.0007) (Figure 4E), and USP29 reconstitution decreased the expression of cleaved caspase-3 compared with the USP29-depleted cells (*p* = 0.0025).

### 2.5. Loss of USP29 Expression Inhibits Colon Cancer Growth In Vitro and In Vivo

**Figure 5:**
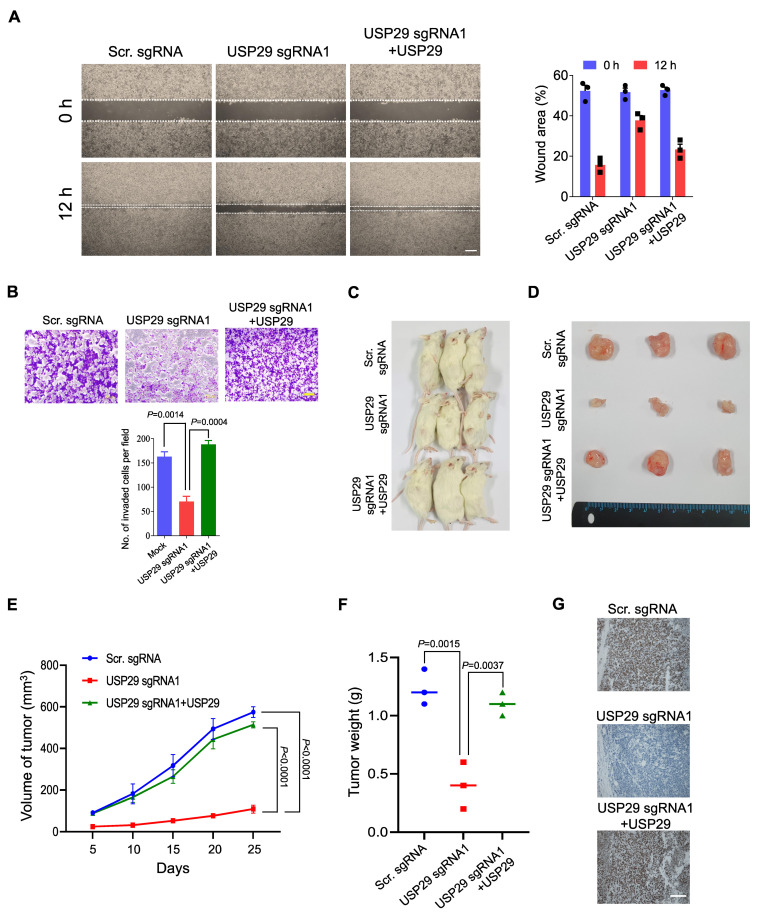
USP29 depletion reduces the pathogenesis of colon cancer in vitro and in vivo. (**A**) HCT116 cells were transfected with the indicated plasmids and wound healing assay was performed to assess for their migratory potential. Representative images were captured at 0 and 12 h (Scale bar = 500 µm) and the percentage of wounded area is indicated in the right panel. (**B**) Matrigel cell invasion assay was performed in HCT116 cells. Cells were transfected with the indicated plasmids and cells were seeded into the upper chamber of the Transwell plate. AT 24 h, cells on the lower side of the chamber membrane were fixed and stained with crystal violet, and cell invasion was quantified graphical representation. Scale bar = 200 µm. (**C**–**F**) HCT116 scrambled sgRNA control, USP29-depleted, and USP29-depleted HCT116 cells with reconstituted USP29 were subcutaneously injected into NSG mice for xenograft experiments. The tumor volume was estimated every other day for 4 weeks and is graphically represented. Then, the tumors were harvested to acquire representative images. The tumor weights were measured and are graphically represented. (**G**) Tumors from the in vivo study was sectioned and embedded in paraffin. IHC was performed using USP29-specific antibody. Scale = 50 µm. Data in (**A**,**B**,**E**,**F**) are presented as the mean ± standard deviation of three independent trials. *p*-values were calculated using Two-way ANOVA.

Because USP29 facilitates colon cancer proliferation, we wondered whether USP29-depleted HCT116 cells might inhibit colon cancer growth in vitro and in vivo. To that end, we analyzed the migration activity of HCT116 cells using a wound healing assay. Compared with the scrambled sgRNA control, the USP29-depleted cells displayed less migration activity, and reconstituting USP29 in USP29-depleted cells recovered their migration potential (Figure 5A).

To further substantiate the oncogenic behavior of USP29, we performed a Matrigel-assisted invasion assay. In line with the previous result, USP29-depleted cells displayed less invasion than the scrambled sgRNA control (*p* = 0.0014), and upon USP29 reconstitution, the number of invading cells increased compared with the USP29-depleted cells (*p* = 0.0004) (Figure 5B).

To further validate the effect of USP29 on oncogenic transformation, we used USP29-depleted HCT116 cells and performed an in vivo tumor formation assay. We subcutaneously injected scrambled sgRNA control, USP29-depleted HCT116 cells, and USP29-depleted cells with reconstituted USP29 into different groups of NSG mice and monitored the tumor growth continuously for 4 weeks. In the NSG mice with USP29-depleted cells, tumor growth was substantially reduced compared to mice that injected the scrambled sgRNA control cells (*p* < 0.0001) (Figure 5C–E). On the other hand, the NSG mice with the USP29-depleted cells with reconstituted USP29 had a substantial increase in the volume of tumor (*p* < 0.0001) (Figure 5E) and weight (*p* = 0.0037) (Figure 5F). Then, xenograft tumors were subjected to IHC analysis to check the status of USP29 expression. USP29 expression was significantly decreased in USP29 sgRNA1 group compared to scrambled sgRNA group. The expression of USP29 was reestablished in the USP29-depleted HCT116 cells reconstituted with USP29 group (Figure 5G). Altogether, our results show that USP29 is highly upregulated in colon cancers and promotes tumorigenesis in vitro and in vivo.

## 3. Discussion

Colon cancer remains a primary cause of cancer-linked deaths worldwide and death rates have been constant for decades [18,19]. A statistical-based investigation estimated that new cases of colorectal cancer have increased from 9.7% to 10.2% of all cancer cases, and the percentage of deaths attributed to colorectal cancer has risen from 8.5% to 9.2% [20,21]. Thus, colon cancer poses formidable challenges to human health, and efforts to develop promising therapeutic regimens are important.

Recent advances in cancer therapies and their prompt application to various cancers have validated the prospects for exploring DUBs as targets for cancer [22,23]. The expressions of various DUBs in several cancers have been well studied [24]. DUBs act by binding to their target proteins, though in some cases, a DUB can be targeted directly for the drug development [25]. Further studies on the cellular localization, expression, and regulation of DUBs will be helpful to comprehend their roles in tumorigenesis and therapeutics. Recently, a study reported that the high expression USP21 was associated with the high-grade CRC and had a significant impact on patients’ life span [26]. Their findings demonstrated that USP21 stabilizes the protein level of activator protein-1 which is previously known to be involved in the CRC progression and metastasis. Similarly, a siRNA-based DUB screening identified PSMD14 as a protein stabilizer of ALK2 [27]. This study showed that the depletion of either ALK2 or PSMD14 resulted in the inhibition of CRC progression and survival of CRC patients. Based on these evidences, targeting DUBs could be an alternative strategy to treat patients with CRC.

In this study, we identified that USP29, an uncharacterized DUB, is overexpressed in CRC. USP29 overexpression increases the proliferative behavior of the HCT116 and SW480 CRC cell lines and has been reported as an indicator of poor prognosis in CRC patients. However, it is unclear that how high USP29 expression correlates with local tumors and further investigation is required on how elevated expression of USP29 is carried over to other solid tumors. Previously, a report suggested that USP29 regulates the Claspin levels and plays a regulatory role in DNA replication [14]. Knockdown of USP29 by siRNA caused DNA damage followed by Chk1 phosphorylation in the U2OS cell line, whereas USP29 overexpression prevented DNA damage. In line with that study, we found that CRISPR-mediated downregulation of USP29 in HCT116 cells significantly activated γH2AX expression. Cells depleted of USP29 displayed defects in progressing through the S-phase. Most important, USP29 knockdown delayed mitosis in HCT116 cells. Recently, a study reported that USP29 depletion decreased apoptosis by regulating the Snail protein [13]. Consistent with those results, loss of USP29 elevated the sub-G1 population and cleaved caspase-3 expression (Figure 4). Furthermore, USP29 depletion in HCT116 cells decreased migration, invasion, and tumor formation (Figure 5). Another study reported that p53 protein was stabilized by USP29 in response to oxidative stress [15]. The induction of USP29 transcription is largely depends on JTV1-FBP expression. It is reported that JTV1 was transported to the nucleus and binds to FBP to activate USP29 transcription. However, the induction of nuclear uptake of JTV1 that causes USP29 activation is highly specific to oxidative stress. Despite the knowledge on the action of DUBs, their substrate specificity and mode of regulation is surprisingly scant. Further study is required to analyze the correlation between USP29 and p53 expression status in CRC and how USP29 is involved in tumorigenesis by deubiquitinating p53 in colon cancer. Altogether, these data indicate that USP29 is a novel DUB that promotes tumorigenesis in CRC, making it a promising therapeutic target for colon cancer.

## 4. Materials and Methods

### 4.1. Cell Culture, Reagents, Plasmids, and Antibodies

HCT116 and SW480 cells were procured from the Korean Cell Line Bank (Seoul, Korea). DMEM (PAN-Biotech, Aidenbach, Germany) was used to maintain the cells in the presence of 10% FBS (Gibco, Waltham, MA, USA) and penicillin-streptomycin (HyClone, Logan, UT, USA) at the concentration of 100 µg/mL. CO_2_ incubator (Thermo Scientific, Waltham, USA) was used to culture the cells at 37 °C. GFP-USP29 and GFP-USP29 C294A (Cysteine to Alanine at 294th position point mutated) plasmids were a gift of Prof. Han Liu (Dalian Medical University, Dalian, China), and Cas9-2A-mRFP-2A-PAC was obtained from Toolgen (Seoul, South Korea). The antibodies used in this study are as follows: USP29 (HPA021064) from Sigma (St. Louis, MO, USA), Ki67 (610969) from BD Biosciences (Franklin Lakes, NJ, USA), γH2AX (05-636) from Millipore (Burlington, MA, USA), phospho-histone H3 (3377) and cleaved caspase-3 (9661S) from Cell Signaling Technology (Danvers, MA, USA), and USP29 (sc-517145), H2AX (sc-517336) and GAPDH (sc-32233) from Santa Cruz Biotechnology (Dallas, TX, USA).

### 4.2. Transfection

HCT116 and SW480 cells were transfected with Lipofectamine 2000 (Invitrogen, Carlsbad, CA, USA) and followed the manufacturer’s recommendations. USP29-depleted HCT116 cells were generated by transfecting Cas9 and sgRNA1 targeting USP29. Post 24-h transfection, fresh media containing 1.5 µg/mL of puromycin was added and selected for 7 days. The USP29 knockout efficiency was validated by qPCR and Western blot analysis. Finally, the depletion of USP29 was confirmed by Sanger sequencing. Similarly, the scrambled sgRNA control was also subjected to puromycin selection as mentioned above and used for the experiments. The scrambled sgRNA sequences are as follows: FP 5′ GCCACGCGTAAGCGGTCCGC 3′ and RP 5′ GCGGACCGCTTACGCGTGGC 3′.

### 4.3. Expression Studies from the TCGA Database

All TCGA expression data were taken from the UCSC Xena browser (https://xenabrowser.net/ (accessed on 9 November 2020)) as processed information (level 3). Gene expression comparisons of normal and tumor groups were analyzed for subjects that have both tumor and matched-normal samples. A paired *t* test was employed to estimate the *p* values.

### 4.4. Immunohistochemistry

Human clinical samples, colon cancer (*n* = 32) and corresponding normal tissues (*n* = 32), were purchased from AccuMax Array (ISU Abxis, Gyeonggi-do, Korea). The formalin-fixed paraffin-embedded tissue samples were subjected to deparaffinization and then incubated with USP29-specific antibody (1:100) as stated by a protocol explained before [28]. The samples were counterstained with hematoxylin followed by dehydration and mounting. Images were made using a Leica model DM5000 B (Leica, Hesse, Germany).

Xenograft samples obtained from in vivo study were embedded in paraffin and sectioned. IHC was performed with USP29-specific antibody (1:100). All images were captured by a Leica DM 5000 B microscope.

### 4.5. Cell Proliferation Assay

The proliferation status of HCT116 and SW480 cell lines was estimated using a Cell Counting Kit-8 (CCK-8) (Dojindo Molecular Technologies, Kyushu, Japan). Post-transfection, CCK-8 solution (10 µL) was added, and incubated at 37 °C for 2 h according to the supplier’s recommendations. Then, absorbance was estimated at 540 nm with microtiter plate reader (Bio-Rad Laboratories, Hercules, CA, USA).

### 4.6. Immunofluorescence

Immunofluorescence was employed to check Ki67 proliferation, DNA double strand break (as measured by γH2AX foci formation), phospho-histone H3 levels, and cleaved caspase-3 activation. We seeded 2 × 10^4^ HCT116 or SW480 cells into a 4-well plate with cover slips and incubated them overnight. Then, the cells were transfected with the indicated plasmids and incubated for 48 h. Subsequently, the cells were fixed with 4% paraformaldehyde (Wako Chemicals, Richmond, VA, USA) at room temperature for 20 min and then washed with Triton-X 100 (0.03%) (Sigma-Aldrich, St. Louis, MO, USA) thrice. 5% BSA (Bovogen Biologicals, Keilor East, Australia) in 1X PBS was used to remove non-specific binding. Blocking was done at room temperature for 1 h followed by primary antibody incubation at 4 °C overnight. Next day, PBS was used to wash the cells and Alexa Fluor–conjugated secondary antibody was added for 1 h at room temperature. DAPI (Invitrogen, Carlsbad, CA, USA) was added at 1 µg/mL concentration to the cells and incubated at room temperature for 15 min to detect the nuclei. Then, the glass slides were used to mount the samples, and a Leica TCS SP5 confocal microscope was used for image acquisition.

### 4.7. Generation of USP29-Depleted HCT116 Cells Using CRISPR-Cas9

HCT116 cell line was co-transfected with sgRNA1 targeting the *USP29* gene and Cas9. After 24 h of transfection, fresh medium containing puromycin (1.5 μg/mL) was added, followed by the selection of transfected cells for 5 days. The concentration of the puromycin was decreased to 0.75 μg/mL, and continued culturing for 10 days. Single colonies were chosen and expanded. Then, a fraction of cells was separated and T7 endonuclease 1 (T7E1) assay was performed to validate the efficiency of *USP29* gene knockout.

### 4.8. T7E1 Assay

The T7E1 analysis was done as mentioned before [29]. In brief, we isolated the genomic DNA (Promega, Madison, WI, USA) and followed the supplier’s instructions. The cas9-edited target region was amplified with the help of hemi-nested PCR and the following primers were used: USP29 first PCR: forward 5′ TCGCCTAACAGCTCCCT TGA 3′, reverse 5′ TAGTGTGTTTTGCCCCTTCCC 3′; second PCR: forward 5′ GAACTGGCACAGCTTCAGAAT 3′ and reverse 5′ TAGTGTGTTTTGCCCCTTCCC 3′. The amplicons generated from the PCR were subjected to heating to denature it and then cooled down to anneal the product for the formation of heteroduplex DNA complex. Afterward, T7E1 (5 units) (Enzynomics, Daejeon, Korea) were added incubated for 20 min at 37 °C. Agarose gel electrophoresis was done, and images were taken in the GelDoc (Bio-Rad Laboratories, Hercules, CA, USA). The occurrence of mutations was assessed using ImageJ software (National Institutes of Health, version 1.51j8, Madison, WI, USA) based on the cleaved product intensity.

### 4.9. Western Blot Analysis

HCT116 cells were washed twice with PBS and harvested. The lysis buffer (1% Triton-X, 150 mM NaCl, 50 mM Tris-HCl pH 8, 1 mM PMSF) was used to lyse the cells and kept on ice for 20 min. Then, the lysed samples were subjected to centrifugation at high speed. The protein was separated from the samples and Bradford reagent (Bio-Rad Laboratories, Hercules, CA, USA) was used to measure the protein concentration. Immuno blot analyses were performed using the following antibodies: Ki67 (1:1000), USP29 (1:1000), γH2AX (1:1000), H2AX (1:1000), and GAPDH (1:2000). The whole western blot figures can be found in the supplementary materials.

### 4.10. Quantitative PCR Analysis

HCT116 cells were transfected with either scrambled sgRNA or sgRNA1 targeting USP29. Cell pellets were collected after 48 h of transfection and total RNA was extracted using TRIzol reagent (Favorgen, Taipei, Taiwan). SuperScript III kit (Thermo Scientific, Waltham, MA, USA) was used to synthesize the cDNA by following the manufacturer’s protocol. Real time PCR system (Thermo Scientific, Waltham, MA, USA) was used to perform the qPCR analysis using the following primers: USP29-F 5′ GGATCTCAAGGAATGGCTGA 3′ and USP29-R 5′ TTCATCTAT GATGCTCTCCTCAAT 3′.

### 4.11. Flow Cytometry

Flow cytometry was performed to assess sub-G1 population by direct measurement of DNA content. Briefly, 2.5 × 10^5^ HCT116 cells per well were seeded and transfected with the indicated plasmids. Collected cells were subjected to cold wash using PBS containing 5% FBS. 70% ethanol was added dropwise to the cells until further use. Then, RNaseA was added at the concentration of 2 mg/mL and incubated for 15 min at 4 °C. Finally, 10 µL of PI at the concentration of 50 mg/mL was added to the cells for 10 min at room temperature to stain the DNA. Finally, flow cytometry (FACS CANTO II, BD Biosciences, Franklin Lakes, NJ, USA) was used to measure the DNA content and the data was analysed using FACSDiva 8.0 software (BD Biosciences, Franklin Lakes, NJ, USA)

### 4.12. Colony Formation Assay

HCT116 cell line was transfected with the plasmids before performing the colony formation assay. 1% agarose and 1×DMEM were mixed together at an equal proportion (*v*/*v*). 35-mm dishes were used to plate above mixture. The transfected cells were seeded (1 × 10^4^) and mixed with 0.7% of DMEM/agarose mixture. After 14 days, 0.01% crystal violet dye in 20% methanol was added for 5 min and incubated at room temperature to stain the colonies. A light microscope was used to capture the images.

### 4.13. In Vitro Scratch Assay

The migratory activity of HCT116 cell line was monitored using the in vitro scratch assay. HCT116 cell line was transfected with the indicated plasmids and were harvested after 48 h. To perform the scratch assay, cells were again seeded at 90% confluence. and Scratch was made on the HCT116 cells using the pipette tip. Each wounded cell layer was washed gently with PBS and incubated in the presence of serum-free DMEM medium. The migrated area was captured under a microscope, IX71 (Olympus Corporation, Tokyo, Japan) at 0 and 24 h respectively. ImageJ software (National Institutes of Health, version 1.51j8, Madison, WI, USA) was employed to estimate the percentage change in the wounded area, as previously described [30].

### 4.14. Matrigel Invasion Assay

HCT116 cells were transfected with the indicated plasmids and invasion was assessed using Matrigel-coated transwell chambers according to the manufacturer’s recommendations. Briefly, HCT116 cells (2.5 × 10^4^) in serum-free DMEM was placed in the upper chamber. Then, the lower chamber was supplemented with the complete DMEM media, and incubated for 24 h at 37 °C. Then, the insert was removed and the lower surface containing the cells was fixed with ice cold methanol at room temperature for 15 min and stained with 0.1% crystal violet. Finally, the invaded cells were quantified using a light microscope, and number of invaded cells was represented graphically.

### 4.15. In Vivo Tumor Formation Assay

We used the 5 weeks old male NOD scid γ mice for the in vivo research. This study was permitted by the IACUC. All mice were maintained in a 12 h light-dark cycle and have the access to water and food. The mice were randomized into three groups (three mice per group) and given one of the following injections subcutaneously into their right flanks: scrambled sgRNA HCT116 cells, USP29-depleted HCT116 cells, or USP29-depleted HCT116 cells with reconstituted USP29 (5 × 10^6^) in an equal ratio of PBS: Matrigel. The volume of the tumors was individually estimated by the two co-authors, who were blinded to the group conditions, every other day for 4 weeks using V = D × d^2^/2, (D = long axis of the tumor and d = short axis of the tumor). Once the experiment is completed, all tumors were collected and weighed, and images were taken.

### 4.16. Statistical Analysis

All statistical analysis was achieved using the GraphPad Prism 9 (San Diego, CA, USA), and data are presented as the mean ± standard deviation of three independent trials. One-way or two-way analysis of variance or Student’s *t*-test was performed. Multiple comparisons between the variables were made using the Tukey’s post-hoc test. *p* < 0.05 was considered as statistically significant difference.

## 5. Conclusions

Our data suggested that USP29 was highly expressed in CRC. Overexpression of USP29 mediates cell proliferation and cell cycle progression of HCT116 cells. CRISPR-Cas9-mediated downregulation of USP29 induces DNA damage and apoptosis in HCT116 cells. USP29-depleted HCT116 cells displayed reduced cell migration and invasion. The tumor xenograft derived from USP29-depleted HCT116 cells showed the reduced volume of the tumor than the scrambled sgRNA control. Altogether, our research highlighted a new therapeutic approach for treating CRC by targeting USP29 signals.

## Data Availability

Not applicable.

## Supplementary Materials

The following are available online at https://www.mdpi.com/article/10.3390/cancers13112706/s1, Figure S1: Endogenous binding between USP29 and Ki67; Table S1: Patient information of TMA were listed. The whole western blot figures can be found in the supplementary materials.
