# Supplementary material for: Ubiquitin Specific Protease 29 Functions as an Oncogene Promoting Tumorigenesis in Colorectal Carcinoma
Source: Cancers (Basel). 2021 May 31;13(11):2706. doi: 10.3390/cancers13112706 (PMC8197902; doi:10.3390/cancers13112706)

Figure S1

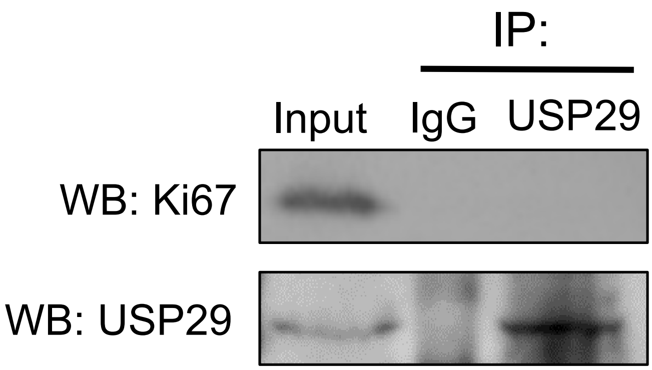

Figure S1: USP29 does not interact with Ki67. HCT116 cells were used to analyze the binding between USP29 and Ki67. IgG was used as a negative control.

Figure S2: Uncropped blots and images used in this study

Uncropped blots of Fig. 2A

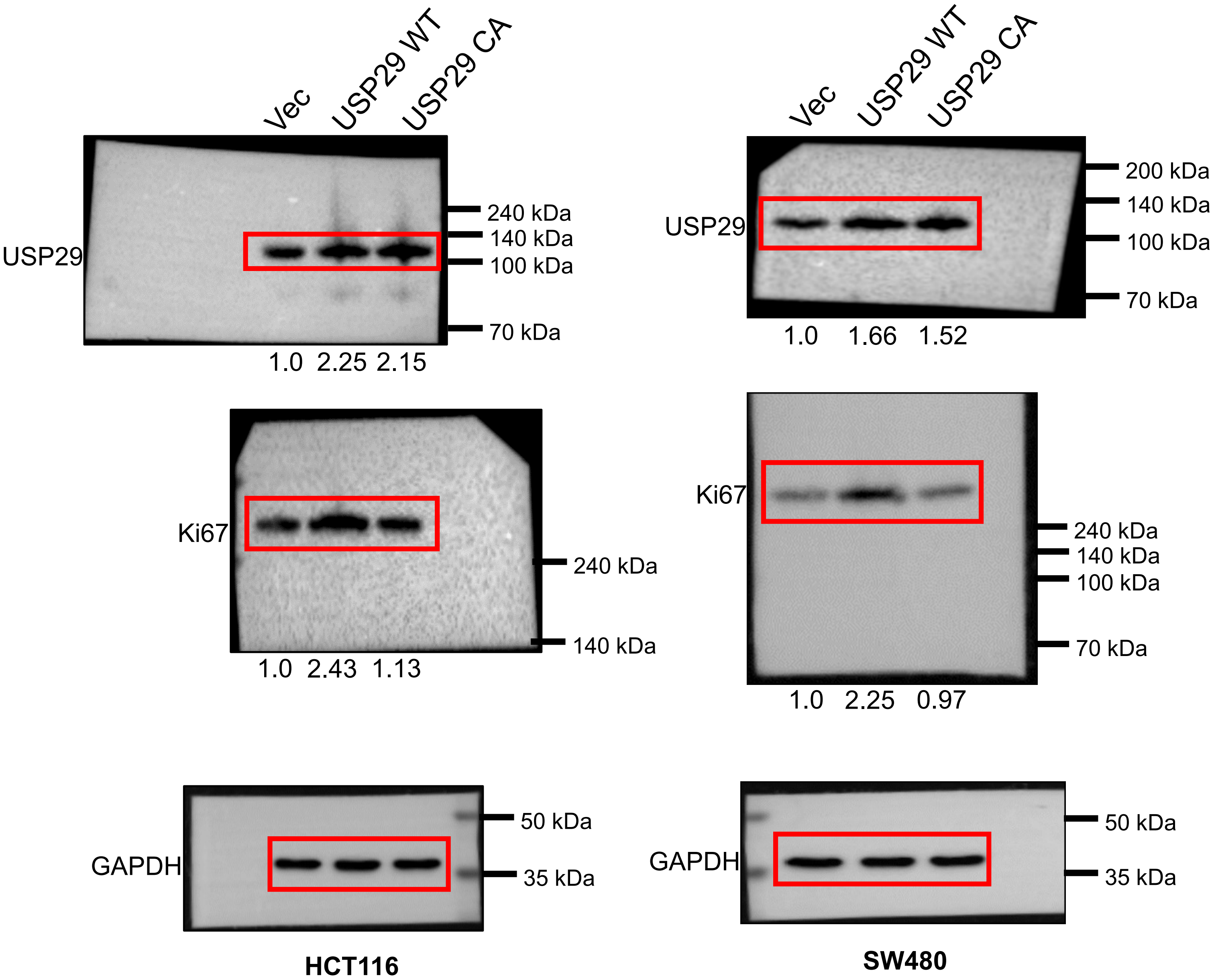

Band intensity ratio (normalized with GAPDH)

Uncropped gel picture of Fig. 2G

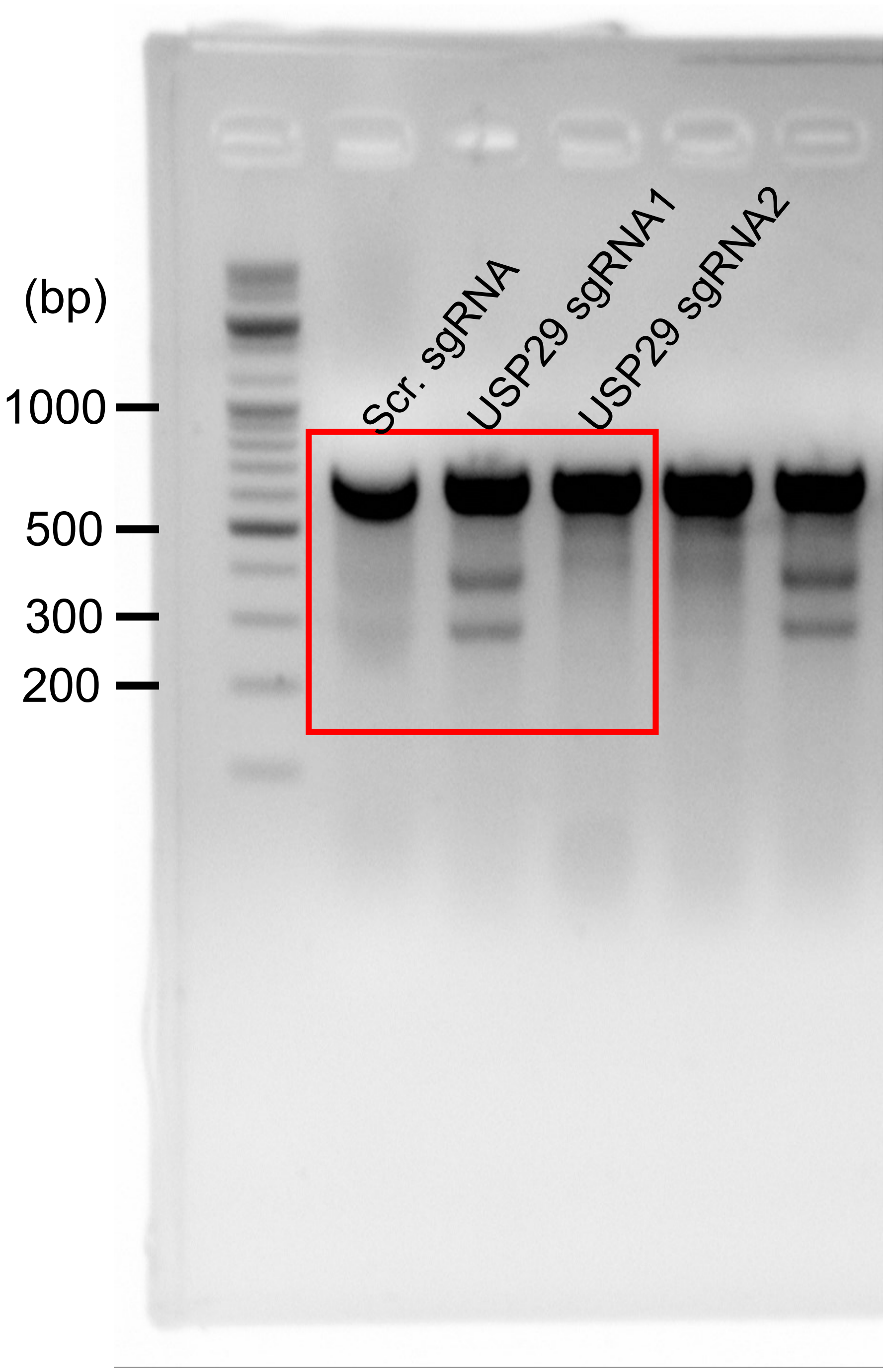

Uncropped blots of Fig. 2J

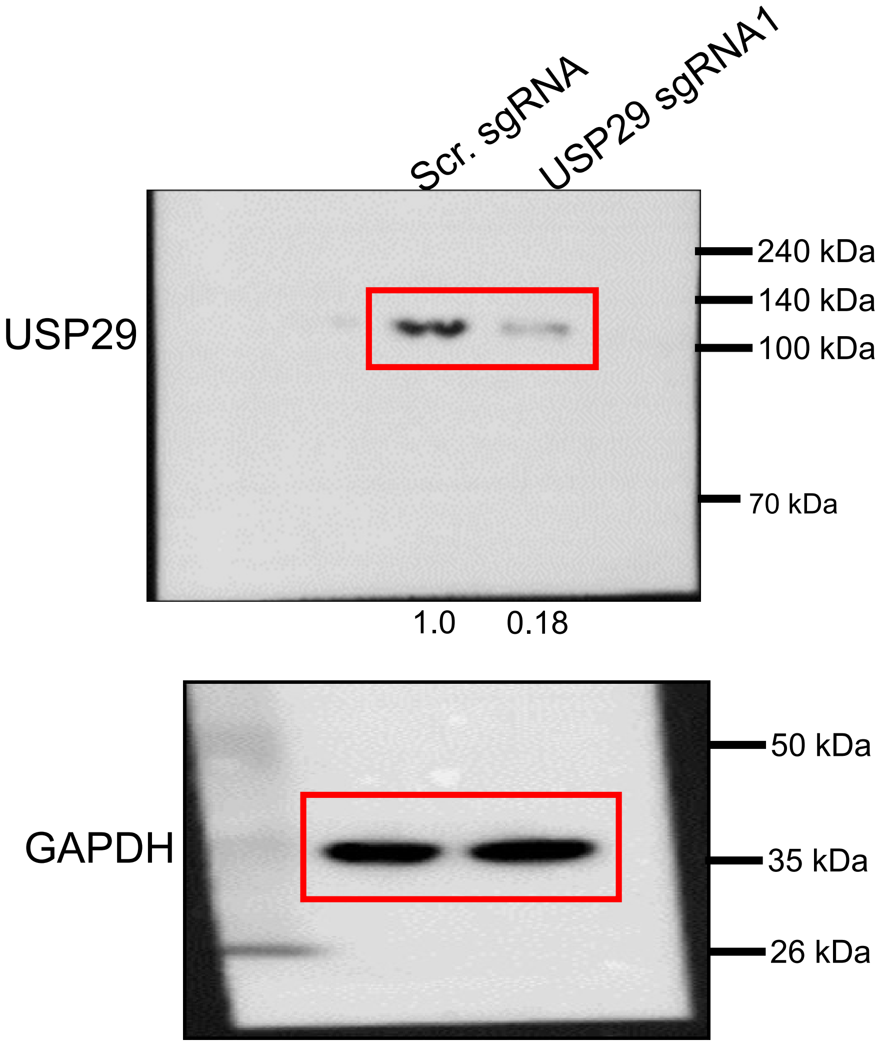

Band intensity ratio (normalized with GAPDH)

Uncropped blots of Fig. 3A

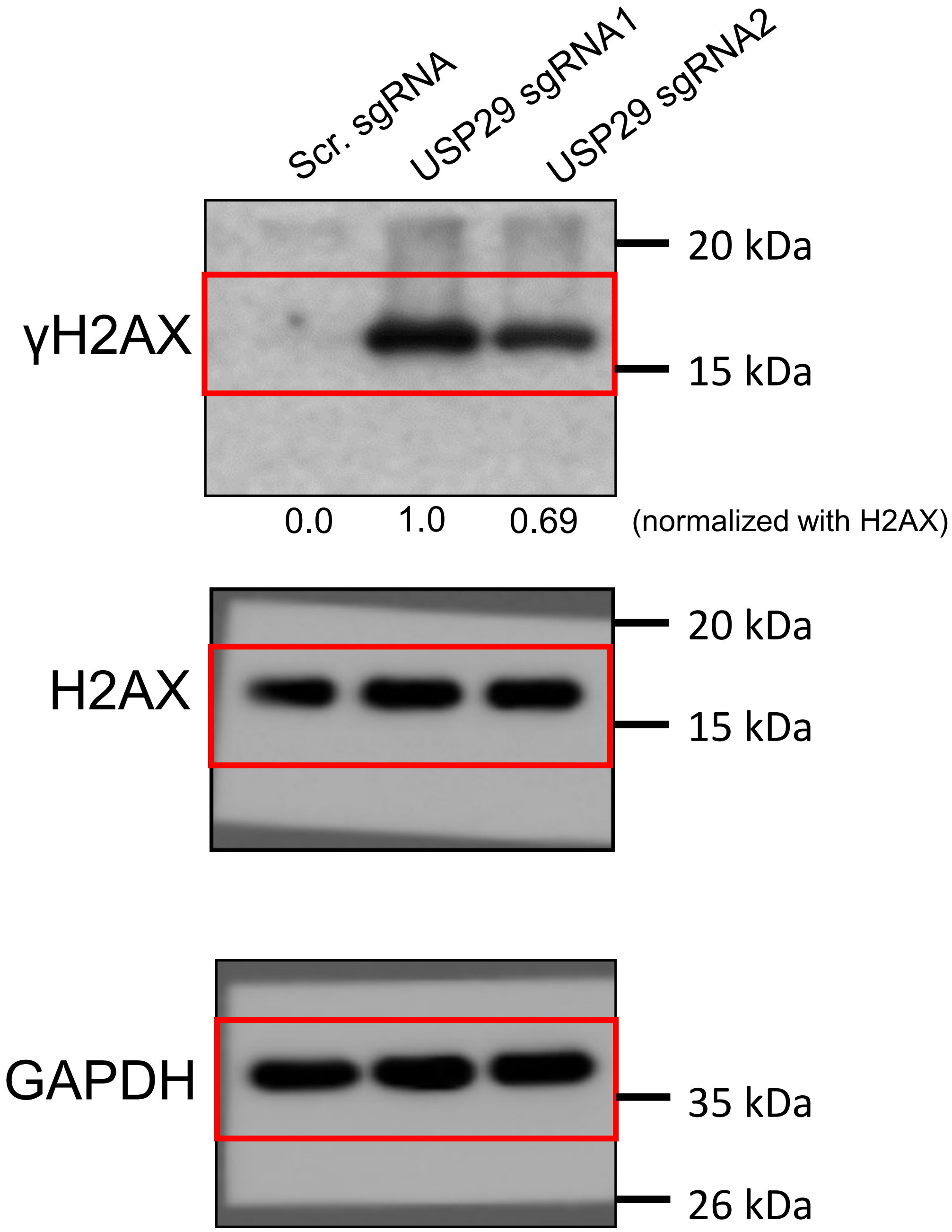

Uncropped blots of Fig. 4A

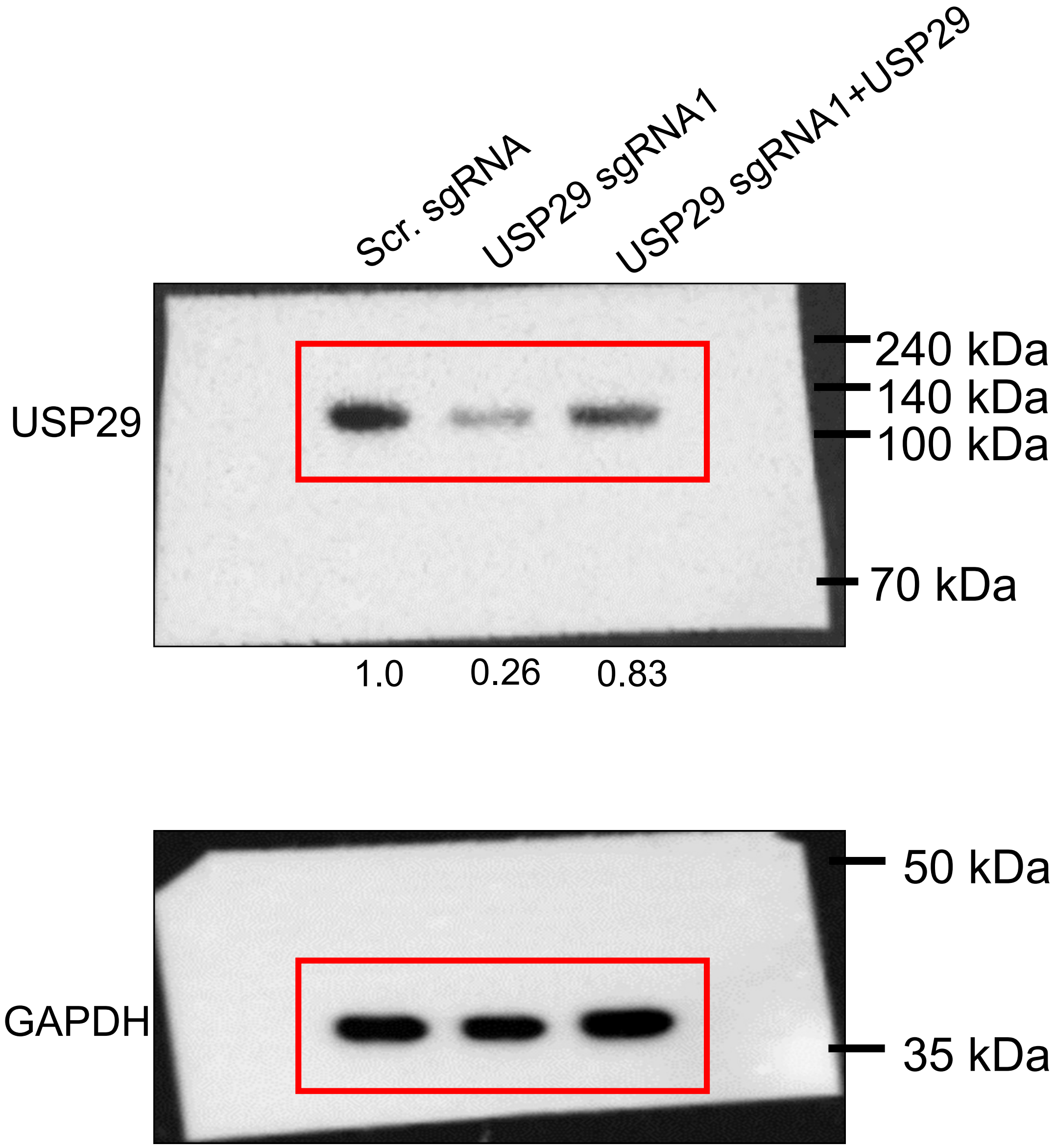

Band intensity ratio (normalized with GAPDH)

Unprocessed images of Fig. 4C

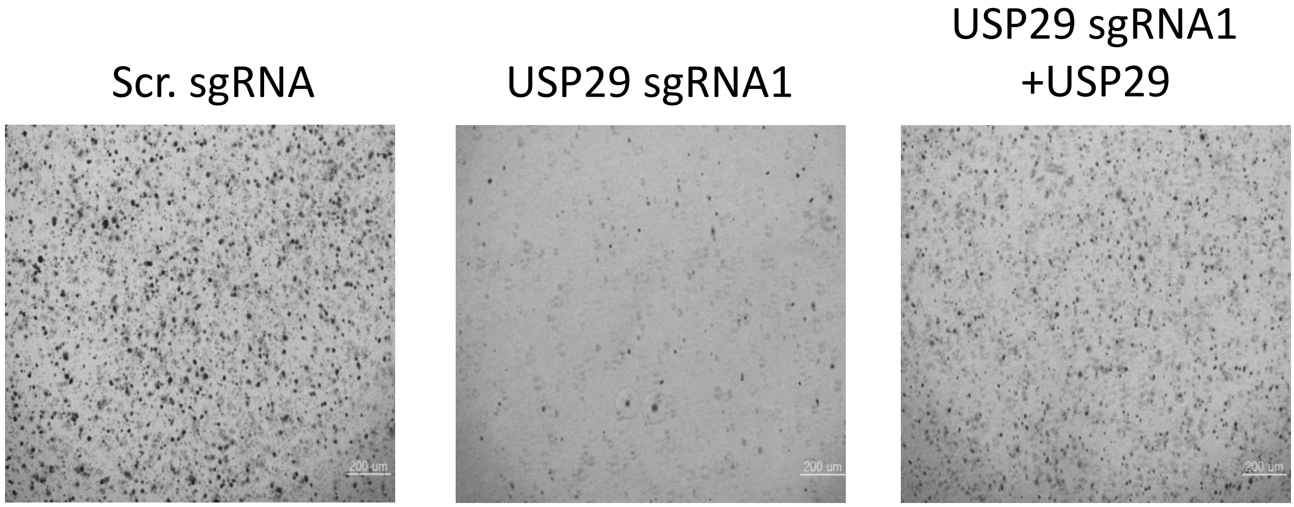

Uncropped blots of Fig. S1

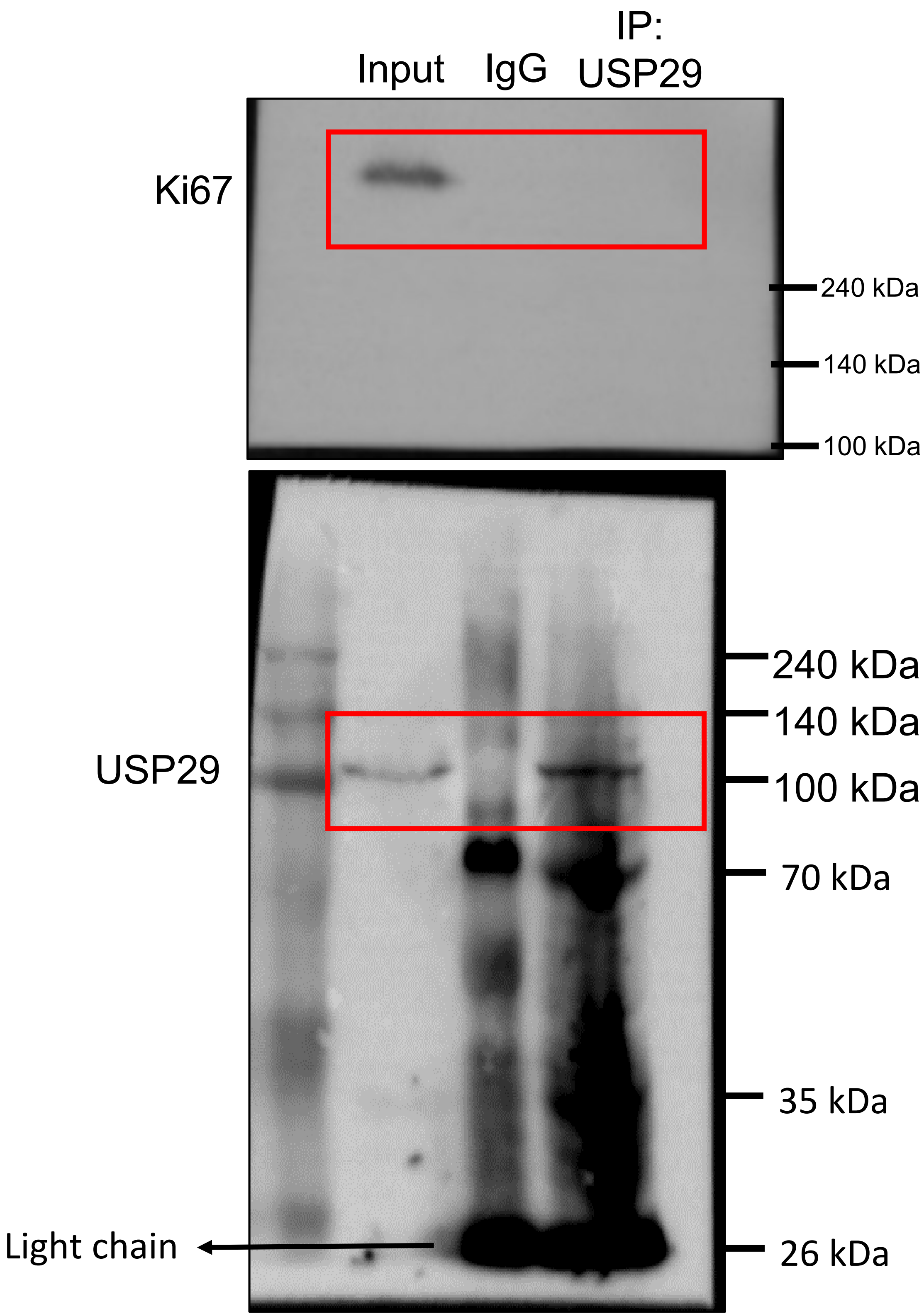

Supplement: Supplementary file 1 [file cancers-13-02706-s001.zip › cancers-1152936-supplementary/Supplementary Figures.pdf]
