# Supplementary material for: Ubiquitin Specific Protease 29 Functions as an Oncogene Promoting Tumorigenesis in Colorectal Carcinoma
Source: Cancers (Basel). 2021 May 31;13(11):2706. doi: 10.3390/cancers13112706 (PMC8197902; doi:10.3390/cancers13112706)
Supplement: Supplementary file 1 [file cancers-13-02706-s001.zip › cancers-1152936-supplementary/Table S1.pdf]

Table S1. Patient information of TMA were listed (Clinicopathological information is not available)

No.	Patient info	Sex	Age	Carcinoma
1	Patient 1	M	82	Adenocarcinoma
2	Patient 2	M	86	Adenocarcinoma
3	Patient 3	M	81	Adenocarcinoma
4	Patient 4	M	81	Adenocarcinoma
5	Patient 5	M	43	Adenocarcinoma
6	Patient 6	M	68	Adenocarcinoma
7	Patient 7	F	78	Adenocarcinoma
8	Patient 8	F	71	Adenocarcinoma
9	Patient 9	F	67	Adenocarcinoma
10	Patient 10	F	70	Adenocarcinoma
11	Patient 11	M	54	Adenocarcinoma
12	Patient 12	M	72	Adenocarcinoma
13	Patient 13	M	69	Adenocarcinoma
14	Patient 14	M	86	Adenocarcinoma
15	Patient 15	M	76	Adenocarcinoma
16	Patient 16	F	84	Adenocarcinoma
17	Patient 17	M	70	Adenocarcinoma
18	Patient 18	M	56	Adenocarcinoma
19	Patient 19	M	90	Adenocarcinoma
20	Patient 20	F	48	Adenocarcinoma
21	Patient 21	F	69	Adenocarcinoma
22	Patient 22	F	75	Adenocarcinoma
23	Patient 23	M	67	Adenocarcinoma
24	Patient 24	F	70	Adenocarcinoma
25	Patient 25	M	63	Adenocarcinoma
26	Patient 26	M	57	Adenocarcinoma
27	Patient 27	M	50	Adenocarcinoma
28	Patient 28	M	67	Adenocarcinoma
29	Patient 29	F	56	Adenocarcinoma
30	Patient 30	F	71	Adenocarcinoma
31	Patient 31	M	58	Adenocarcinoma

32	Patient 32	F	52	Adenocarcinoma
